# Pathophysiology of chronic subdural haematoma: inflammation, angiogenesis and implications for pharmacotherapy

**DOI:** 10.1186/s12974-017-0881-y

**Published:** 2017-05-30

**Authors:** Ellie Edlmann, Susan Giorgi-Coll, Peter C. Whitfield, Keri L. H. Carpenter, Peter J. Hutchinson

**Affiliations:** 1 Division of Neurosurgery, Department of Clinical Neurosciences, University of Cambridge, Box 167, Cambridge Biomedical Campus, Cambridge, CB2 0QQ UK; 2 Southwest Neurosurgical Centre, Plymouth Hospitals NHS Trust, Plymouth, PL6 8DH UK

**Keywords:** Angiogenesis, Chronic subdural haematoma, Inflammation, Head injury, Drug therapy

## Abstract

Chronic subdural haematoma (CSDH) is an encapsulated collection of blood and fluid on the surface of the brain. Historically considered a result of head trauma, recent evidence suggests there are more complex processes involved. Trauma may be absent or very minor and does not explain the progressive, chronic course of the condition. This review focuses on several key processes involved in CSDH development: angiogenesis, fibrinolysis and inflammation. The characteristic membrane surrounding the CSDH has been identified as a source of fluid exudation and haemorrhage. Angiogenic stimuli lead to the creation of fragile blood vessels within membrane walls, whilst fibrinolytic processes prevent clot formation resulting in continued haemorrhage. An abundance of inflammatory cells and markers have been identified within the membranes and subdural fluid and are likely to contribute to propagating an inflammatory response which stimulates ongoing membrane growth and fluid accumulation. Currently, the mainstay of treatment for CSDH is surgical drainage, which has associated risks of recurrence requiring repeat surgery. Understanding of the underlying pathophysiological processes has been applied to developing potential drug treatments. Ongoing research is needed to identify if these therapies are successful in controlling the inflammatory and angiogenic disease processes leading to control and resolution of CSDH.

## Background

**Fig. 1:**
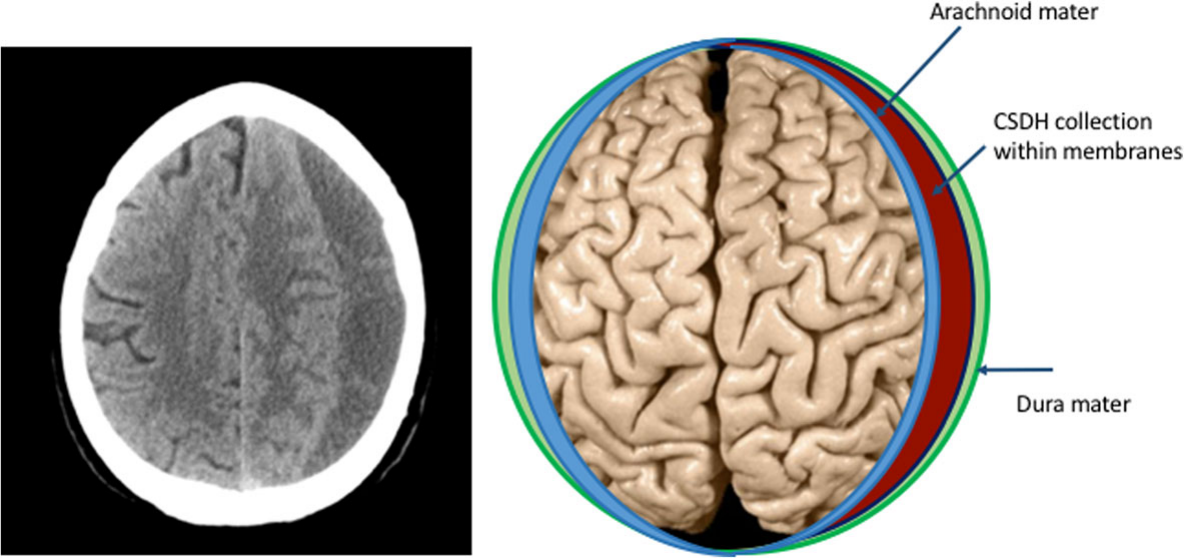
Computed tomography (CT) head scan and schematic representation of a CSDH

Chronic subdural haematoma (CSDH) is an encapsulated collection of fluid, blood and blood degradation products layered between the arachnoid and dura mater coverings on the brain’s surface (Fig. 1). An early theory about the formation of CSDH was of a traumatic injury causing tearing of the bridging veins traversing from the brain to the draining dural-venous sinuses [1, 2]. This in turn would result in the accumulation of venous blood within the subdural space over time, but this theory has long been disputed and for good reason. Firstly, it is well recognised that it takes a mean of 4 to 7 weeks following trauma for a CSDH to become symptomatic [3, 4]. A slow venous haemorrhage from the outset would accumulate sufficiently quickly to cause a symptomatic collection within days. Modern-day easy-access imaging also allows the majority of patients to be scanned at the time of acute trauma. These scans can be entirely normal, with no sign of haemorrhage, but the patient can still go on to develop a CSDH weeks to months later. The pattern of blood seen in CSDH, spanning the cerebral convexities, is also inconsistent with a source of bleeding from a bridging vein, which neighbours the medial venous sinuses [5]. Finally, although CSDH can contain areas of acute haemorrhage, many are almost entirely “old” haematoma, seen as homogenous hypodensity on CT (Fig. 1). Despite this, the collections continue to enlarge over time, suggesting that acute haemorrhage is not the only source of growth.

It has been hypothesised that inflammation is a key factor in the development of a CSDH. This is not a new concept and indeed one of the earliest reports by Virchow in 1857, referred to the condition as “pachymeningitis haemorrhagica interna” [6]. This was based on the assumption that bacterial infection (meningitis) was driving a chronic inflammatory response in the dura, resulting in fibrin exudation and growth of new capillaries. However, it has long become accepted that inflammation can occur in response to any injury, including trauma or cellular injury, and not just infection. The primary purpose of inflammation is for the body’s immune system to activate repair, but despite this intention to heal, persistent or chronic activation of inflammation can occur and lead to pathology. This is exemplified by conditions such as rheumatoid arthritis and asthma, where inflammatory cells contribute to joint destruction or airway oedema and remodelling, respectively [7, 8].

The large number of reports linking CSDH to trauma clouded this early insight regarding inflammation and led to the popular view that CSDH was an entirely traumatic condition [9]. However, some authors did recognise that inflammation and trauma may be co-existing factors in CSDH development and that the trauma need only be very trivial [10].

In 1946, Inglis published an important summary of the key features required for CSDH formation [11]. Following histological analysis of several cases, he identified that the dura is lined with a layer of specialised, modified connective tissue cells. These cells have two essential roles: they can phagocytose, and they can develop into fibro-cellular connective tissue, allowing formation of new membranes as seen in CSDH. These cells have latterly been named “dural border cells” and are considered the location in which a CSDH initially develops [12]. Inglis reported that haemorrhage in the subdural space was sufficient to initiate the proliferation of these dural border cells [9]. Many animal studies have shown that the presence of blood alone, in the subdural space, is not sufficient to initiate progression to a CSDH, although these models are limited by the nature that animals do not have other contributing factors found in human patients, such as cerebral atrophy [5, 13, 14]. However, we do know this to be true in humans as well, as shown by a study on 38 acute subdural haematoma patients where only eight (21%) progressed to form CSDHs [15]. Therefore, it is clear there is more to this condition than trauma causing bleeding into the subdural space.

**Fig. 2:**
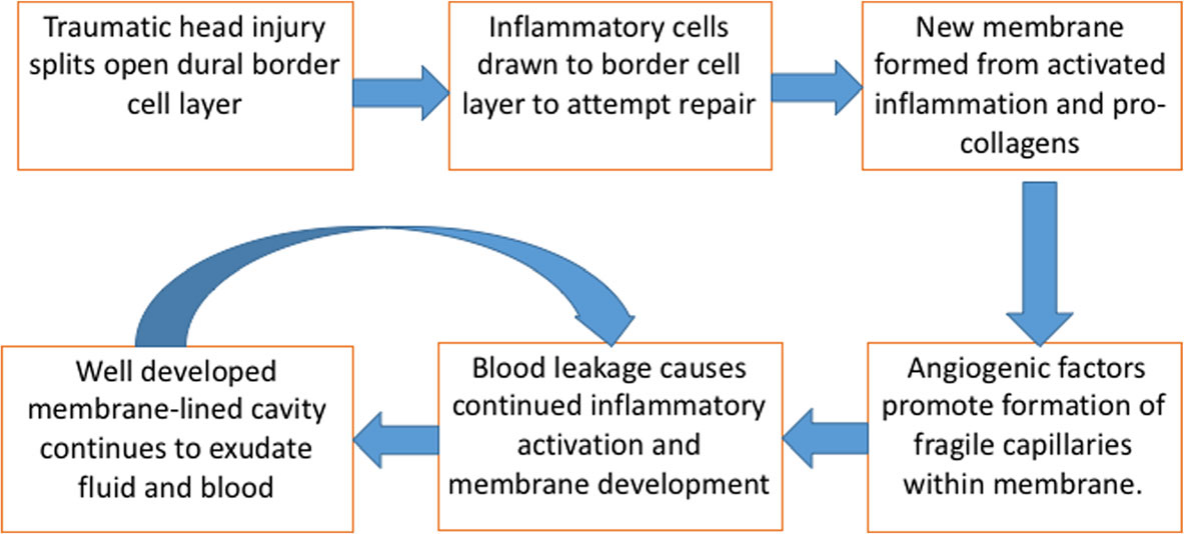
The CSDH cycle. Summary of the pathophysiological processes involved in the formation of a CSDH

Whilst inflammation can aid tissue repair, it is the *sustained* inflammatory response in CSDH which results in new membrane growth and fluid accumulation over time. Damage to the dural border cells, rather than acute haemorrhage itself, may be what initiates this inflammatory response and may not occur in all patients. Inflammatory cells recruited to the subdural space will attempt to repair the border cell damage, but instead proliferate and result in the new membrane formation. Many of the inflammatory cells have pro-angiogenic roles which support the development of new blood vessels in this subdural region. These vessels are “leaky”, allowing micro-haemorrhages and fluid exudation into the new membrane-bound subdural space. This process is summarised in Fig. 2, the so-called CSDH cycle.

Following the pathological delamination of the dural border cells, two membranes are formed, enclosing the new subdural cavity which fills with the fluid and blood. High concentrations of type 1 (PICP) and type 3 (PIIINP) procollagen are present in this fluid and suggest a fibro-proliferative process is occurring, similar to that seen in wound healing [16, 17]. This process is likely to be initiated when the dural border cells are separated, and is an attempt at a reparative process. However, the collagen synthesis outweighs its breakdown, leading to the continuous formation of a collagen matrix and hence formation of the new membranes [17]. The SMAD signalling pathway is an important mediator of the persistent fibrosis which contributes to membrane development, and is activated by transforming growth factor (TGF-β1), expressed by eosinophils [18]. The internal and external membranes are subsequently formed, which relate to the layers contiguous with the arachnoid and dura mater, respectively.

The internal membranes are generally reported as containing collagen and fibroblasts only and therefore relatively non-functional with respect to driving CSDH growth [19]. One histological study using a scanning electron microscope (SEM) did identify occasional blood vessels in the internal membrane, but even these seem to disappear with maturation of the CSDH [20]. The external membrane, however, is considered more crucial in driving CSDH growth. It contains layers of fibroblasts and collagen fibres with the addition of inflammatory cells such as neutrophils, lymphocytes, macrophages and eosinophils [20–25].

Importantly, CSDH membranes also contain numerous highly permeable capillaries with thin walls containing thin or absent basement membrane and lacking smooth muscle cells and pericytes [20, 26]. Gap junctions are also numerous, allowing continued migration of erythrocytes, leucocytes and plasma from these vessels into the subdural haematoma cavity [19, 26]. The external membrane also shows evolutionary changes over time with progressive inflammation followed by scarring, and persistence of areas capable of recurrent bleeding [20]. Importantly, the outer membranes are a well-recognised source of mediators that drive inflammation and angiogenesis such as tissue plasminogen activator (tPA), thrombomodulin, angiopoietin-2 (Ang-2), vascular endothelial growth factor (VEGF) and matrix metalloproteinases (MMPs) [23, 27–30].

**Fig. 3:**
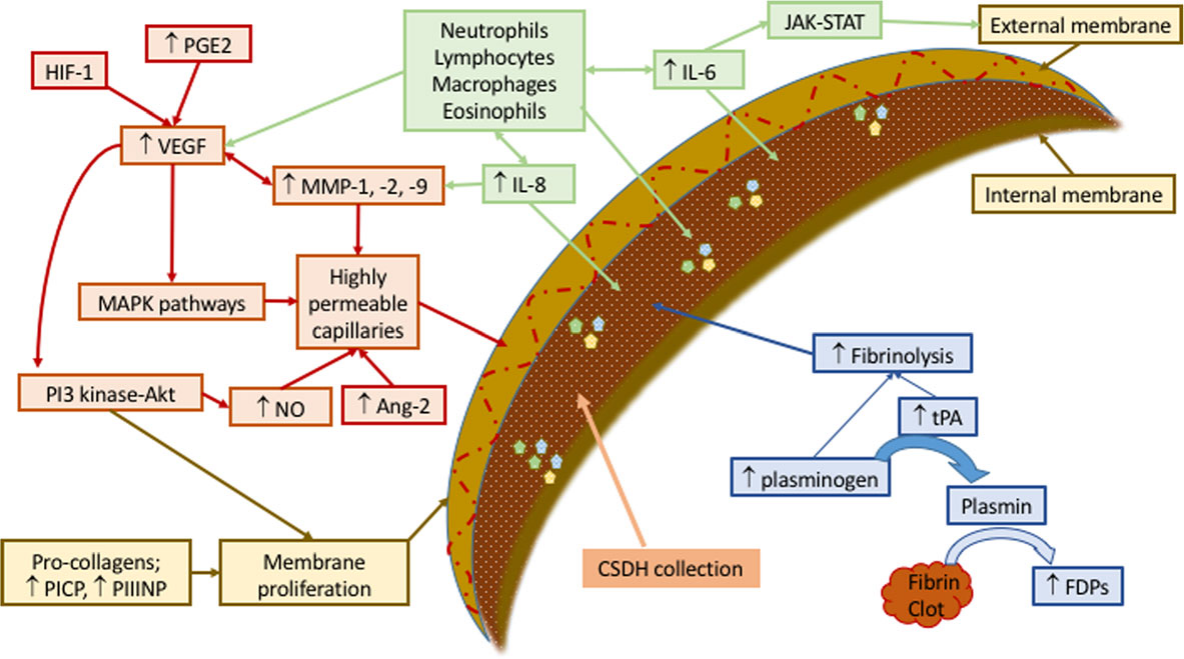
Summary of molecules associated with CSDH formation including recruitment of inflammatory cells (*green*), angiogenesis of highly permeable and leaky capillaries (*red*), processes supporting membrane formation (*brown*) and fibrinolysis promoting further haemorrhage (*blue*). *Abbreviations: Ang* angiopoietin, *FDPs* fibrin/fibrinogen degradation products, *HIF* hypoxia-inducible factor, *IL* interleukin, *JAK-STAT* Janus kinase-signal transducer and activator of transcription, *MAPK* mitogen-activated protein kinase, *MMP* matrix metalloproteinase, *NO* nitric oxide, *PGE* prostaglandin E, *PI3-Akt* phosphatidylinositol 3-kinase-serine/threonine kinase, *PICP* procollagen type 1, *PIIINP* procollagen type 3, *tPA* tissue plasminogen activator, *VEGF* vascular endothelial growth factor

The inflammatory process involved in CSDH membrane and fluid formation is localised to the subdural space, exemplified by the fact that the mediators (in Table 1 and Fig. 3) are consistently significantly higher in and around the CSDH compared with peripheral blood. Each of these mediators may have a unique and important role in CSDH formation and propagation. The following review will discuss recent findings in the literature relating to the cells and markers of inflammation, angiogenesis (formation of new blood vessels) and fibrinolysis (clot breakdown) and how understanding of these processes can help us develop pharmacological therapies for the future. There are several other markers and cell types not directly related to inflammation and angiogenesis and not discussed at length in the present review, but which may still have potentially important roles. These include aquaporin-1, a water channel, heavily expressed in the outer membranes which may contribute to fluid accumulation in CSDH growth [31]; high levels of linoleic acid residue, which have been found in association with recurrent CSDH [32] and a wide range of proteins, similar to those found in serum, suggesting exudation from the outer membrane vessels [33, 34]. CSF is also highlighted as a potential driver of CSDH formation, accumulating due to arachnoid tearing at the time of trauma and potentially continuing to leak into the subdural space. Ninety-three per cent of CSDH collections were reported as positive for CSF, as measured by βTP (beta trace protein), with highest levels seen in patients with recurrent CSDH [35].

**Table 1:** Key mediators in CSDH pathophysiology

Mediator	Finding in CSDH
Type 1 and type 3 procollagen	High levels in CSDH fluid signify fibro-proliferation occurring in CSDH which may relate to neomembrane formation [16, 33].
Thrombomodulin, TPA, fibrin and FDPs	Raised in CSDH fluid and signify hyperfibrinolytic activity occurring [28, 39–41, 74].
Angiopoietin-2	Pro-angiogenic factor, mRNA in high levels in outer membrane of CSDH [29].
VEGF	Pro-angiogenic factor. Very high levels in CSDH fluid and mRNA in membranes and neutrophils [21–23, 29, 46–49, 53].
PGE2	Regulates VEGF. High levels in CSDH fluid correlate with time from trauma [21].
HIF-1α	Regulates VEGF. High staining in outer membrane of CSDH [23].
MMP-1, -2 and -9	Present in outer membrane and CSDH fluid. Contributes to poor capillary integrity [30, 48, 57].
Cytokines and chemokines	High levels of IL-6, IL-8, IL-10, TNF-α, MCP-1, eotaxin-3, CXCL9 and CXCL10 in CSDH fluid compared with serum [18, 21, 53, 61, 62, 73, 74, 80, 83].

### Clinical relevance

**Fig. 4:**
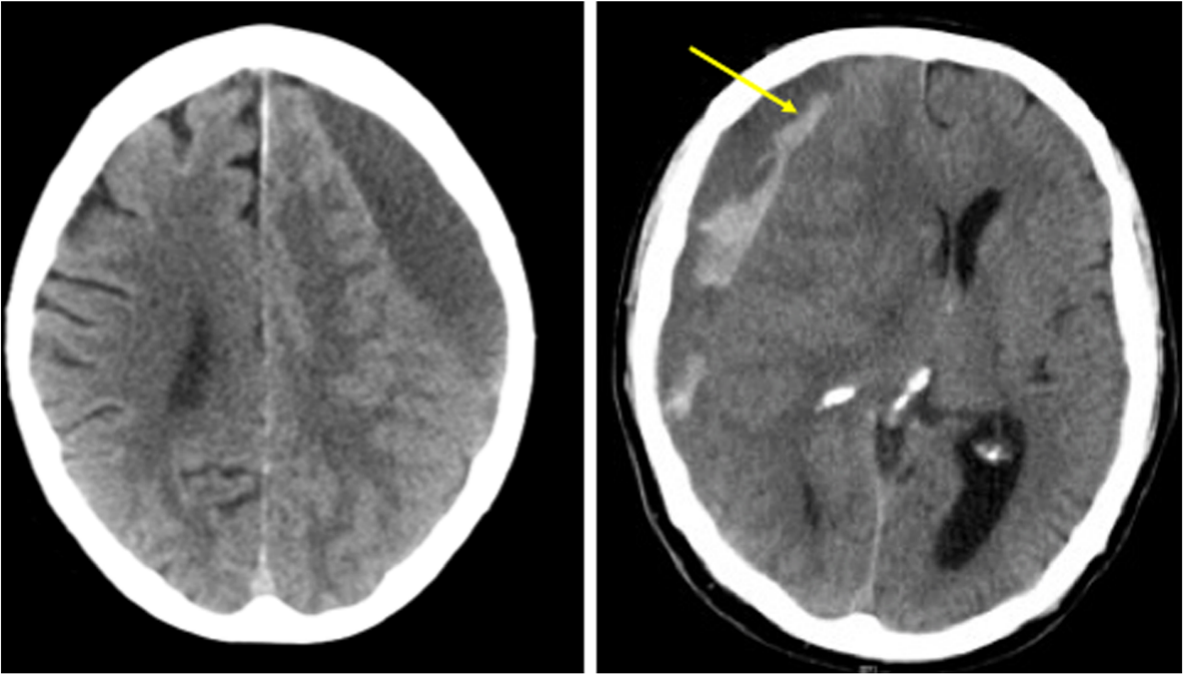
Different patterns of CSDH: *left image* represents a more chronic, hypodense collection, whilst *right image* shows hyperdensity (see *arrow*), representing fresh bleeding

CSDHs can vary in their appearance on imaging, with more hypodense (darker) collections on CT being considered more chronic and those with hyperdensity (brightness) signify more recent or acute areas of haemorrhage (Fig. 4). The CSDH membranes can be correlated with imaging subtypes, which can in turn relate to the clinical presentation. More mature membranes (e.g. with scar features) are correlated with more hypodense, and hence chronic, haematomas [36], whilst more immature membranes exhibit more exudation and bleeding (hyperdensity) and are seen in patients who present with a worse clinical state. It is postulated that more immature membranes represent an earlier stage in the inflammatory cycle and correlate to a more rapid stage of expansion and hence worse clinical presentation [36]. Patients with more mature membranes have already passed through this phase and show larger but more stable haematomas with a more gradual onset of symptoms. Therefore, membrane patterns and histology may help with aging and understanding the growth patterns in CSDH. It is not surprising that there are different periods of inflammatory cycle and intensity, although how this knowledge can be used to guide treatment requires further study.

## Fibrinolysis

It is evident that bleeding is an essential part of CSDH formation and this involves two key biomolecular processes: coagulation and fibrinolysis. Activation of the coagulation cascade involves converting prothrombin to thrombin, the latter of which can then cleave fibrinogen into fibrin, allowing clot formation [37]. Fibrinolysis is the converse process, whereby fibrin is broken down into fibrin/fibrinogen degradation products (FDPs) and clot breakdown-facilitated. Plasmin is the primary mechanism by which fibrinolysis occurs, and it is formed from plasminogen by tissue plasminogen activator (tPA) [37].

The high levels of FDPs which have been found in CSDH fluid are thought to represent excessive fibrinolysis (clot breakdown) and hence continued haemorrhage [33, 38, 39]. One study clearly identified fresh red cells within CSDH fluid, suggesting new haemorrhage occurs [38]. Patients were transfused with labelled red blood cells 24 h before surgical drainage of the CSDH, which estimated daily haemorrhage rates to be 10% on average. The highest levels of new haemorrhage were seen in those patients who showed recent clinical deterioration, suggesting this new bleeding is likely to contribute to CSDH expansion. Very high levels of FDPs were also identified and correlated with the amount of recent haemorrhage; this may suggest fibrinolysis encourages rebleeding. The levels of fibrin and FDPs are also higher in certain CSDH subtypes: mixed density and layered [39]. This may signify different bleeding frequency in CSDH over time, causing haematoma growth to vary and different patterns to form.

Higher levels of plasminogen at initial operation have been identified in patients subsequently experiencing CSDH recurrence [40]. This suggests that markers of hyperfibrinolysis may be able to predict those at highest risk of recurrence and therefore help guide treatment/follow-up. Similar findings have been seen, with high levels of tPA at initial surgery indicating a high risk of recurrence [41]. The tPA has been shown to be produced by blood vessel walls in the CSDH external membrane, and soluble tPA can diffuse from here into the CSDH cavity [27]. Thrombomodulin is another substance which can enhance fibrinolysis, and has been found in high levels in CSDH fluid [28]. Vessel endothelium in the CSDH external membrane was positive for the thrombomodulin antigen in 93% of patients, again suggesting this as the source. It was also suggested that thrombomodulin is continuously secreted in CSDH formation due to repeated vessel injury, possibly even from the brain pulsations through the CSDH collection.

Paradoxically, this hyperfibrinolytic mechanism can also be exploited during surgical evacuation of CSDH with tPA used as an adjuvant therapy [42]. The theory is that the tPA liquefies haematoma further, aiding washout of clots, reflected by substantially increased post-operative drainage volumes and significantly lower recurrence rates. However, this study only reviewed 15 patients treated with tPA, and therefore, this treatment is not well validated.

## Angiogenesis

Many of the mediators related to CSDH formation have a primary role in helping the formation of new vessels (angiogenesis) and therefore provide a bleeding source from the CSDH membranes.

### Angiopoietins

Angiopoietins (Ang) are a group of growth factors involved in regulating angiogenesis and vascular permeability. They have been reported to play an important role in permeability of normal brain membranes, the so-called blood-brain barrier (BBB), and therefore, it is unsurprising that they may also be implicated in CSDH membrane permeability [43]. The two ligands, Ang-1 and Ang-2, have the same tyrosine kinase receptor (Tie-2) but opposing effects on angiogenesis [44]. Higher levels of Ang-1 are present in mature vascular beds with stable microvascular networks and low permeability, as seen in normal brain tissue [29]. In contrast, the CSDH outer membrane has been shown to over-express Ang-2 messenger RNA (mRNA), which is likely to represent ongoing angiogenesis with destabilisation of blood vessel structure [29]. Hence, Ang-2 over-expression may be one of the driving forces for the new, fragile vessel formation in the CSDH membrane.

### Vascular endothelial growth factor and associated factors

Vascular endothelial growth factor (VEGF) is a family of seven growth factors which are the most important pro-angiogenic factors, involved in potentiating microvascular permeability [45]. All members of the VEGF family share a common domain and share two tyrosine kinase receptors (VEGF-R), and for the purpose of this report, we will consider them as one factor [45].

There is a wealth of evidence that VEGF and VEGF-R are found in significantly higher concentrations in CSDH fluid compared with peripheral blood and CSF [21–23, 29, 46–48]. The difference in haematoma levels of VEGF can be more than 28 times that found in serum, with mean serum levels in 20 CSDH patients of 355 pg/mL and CSDH levels of 10,277 pg/mL [22].

The source of this growth factor is debated, but studies have suggested it may be produced by neutrophils from within the CSDH fluid, infiltrating macrophages or the vascular endothelial cells within the CSDH membrane [22, 23, 29, 49]. The downstream signalling pathways mediated by VEGF have been investigated and may include the mitogen-activated protein kinase (MAPK) pathways: Ras-Raf-MEK-ERK, p38 and c-Jun N-terminal kinase (JNK) [50, 51]. These signalling molecules have been identified in the endothelial cells and fibroblasts of CSDH outer membranes and play an important role in disruption of endothelial gap junctions and regulation of angiogenesis [50, 51]. Another signalling pathway which is activated by VEGF in the endothelium of outer membrane vessels is the phosphatidylinositol 3-kinase-serine/threonine kinase (PI3-Akt) pathway [52]. This pathway is involved in production of nitric oxide (critical in angiogenesis) and monitors cell proliferation, permeability and repair [52]. Therefore, an excess of VEGF is capable of inducing angiogenesis and excessive vascular permeability, which may contribute to the ongoing rebleeding implicated in CSDH growth [22]. This is supported by correlations between VEGF concentrations and the CSDH imaging subtypes which are thought to reflect increased rebleeding [23, 48]. Further to this, higher levels of VEGF expression in the outer membrane have been associated with a higher probability of CSDH recurrence [53].

There are other factors directly related to VEGF which are also important. Prostaglandin E (PGE2) is synthesised from arachidonic acid by cyclooxygenase (COX)-2, which regulates VEGF expression [45]. High PGE2 levels have been found in CSDH fluid and are correlated with the time interval since trauma [21]. Therefore, PGE2 may be a good correlate for the escalating inflammatory process occurring over time. Hypoxia-inducible factor 1α (HIF-1α) is a transcription factor integral to the body’s response to hypoxia, which also regulates VEGF and VEGF-R gene expression [45]. Positive staining for this factor has been found in CSDH membranes and correlates with VEGF concentration, making it another potential target for regulating angiogenesis. [23].

Although VEGF has a pro-angiogenic and potentially pro-inflammatory role, it also has a contrasting role in promoting wound repair and neuroprotection [43, 45]. It has also been shown to have a role in neurogenesis and aid recovery in traumatic brain injury (TBI) [54]. This exemplifies the paradoxical roles some mediators have in both potentially harmful inflammation and beneficial repair mechanisms, making them a challenging therapeutic target.

### Matrix metalloproteinases

Matrix metalloproteinases (MMPs) are a family of proteolytic enzymes responsible for digesting the extracellular matrix and are released by endothelial cells during the early stages of vessel growth [55]. They play a critical role in angiogenesis, and their inhibition leads to suppressed angiogenic response with fewer and shortened blood vessels [55]. Increased MMP expression is seen in almost all human diseases involving inflammation and contributes to the inflammatory processes by modulating other mediators (cytokines and chemokines) [56]. MMP proteolysis of endothelial cell junctional proteins also alters barrier permeability, which aids the infiltration of inflammatory cells to otherwise privileged compartments [56]. This is shown with increased vascular permeability or BBB permeability. High MMP-9 and MMP-2 expression has been identified in haemorrhagic brain tumours with associated basement membrane disruption [57]. It has been suggested that these MMPs, alongside VEGF, are responsible for the instability of newly formed blood vessels in tumours, leading to a higher risk of haemorrhage. High levels of MMP-9 have also been identified in the peripheral blood, CSF and peri-contusional brain fluid in acute TBI patients and may contribute to BBB breakdown and subsequent brain oedema [58–60].

Two studies examining MMPs in CSDH membranes and fluid have highlighted MMP-1, -2 and -9 as being present and likely factors contributing to the formation of fragile, leaky capillaries [30, 48]. These in turn are likely to contribute to CSDH growth by allowing haemorrhage and exudation of fluid from the capillaries into the haematoma cavity. The levels of MMP-2 and -9 also correlate with VEGF concentration, suggesting a combined angiogenic process [48].

## Inflammation

As previously discussed, inflammation in CSDH appears to be mediated by a range of inflammatory cells including neutrophils, lymphocytes, macrophages and eosinophils [20–25]. These inflammatory cells both produce and are activated by cytokines: small, inducible proteins. Different families of cytokines are described, based on their structure and receptor type, and chemokines are a subfamily specifically involved in leucocyte recruitment. It is difficult to know the relevance of each individual cytokine as it is well recognised that they work in cascades, influence one another, can compete for receptors and act antagonistically or synergistically. A gross analysis of pro- and anti-inflammatory cytokines in CSDH shows they are both raised in CSDH fluid compared with serum, but that the balance is significantly more pro- than anti-inflammatory [61, 62]. It is important to consider the balance of both the pro- and anti-inflammatory molecules and how this changes over time. Inflammatory markers are not always detrimental, and certain markers which are perceived to be harmful may be required in reparative processes at later time points.

### Interleukin-1

Interleukin-1 (IL-1) was the first cytokine to be discovered, and although 11 different molecules compose the family (IL-1α, IL1-β, IL-18, IL-33, IL-36α, IL-36β, IL-36γ, IL-1Ra, IL-36Ra, IL-38, IL-37), often only IL-1α and -1β are considered when referring to IL-1 [63]. These two ligands have very similar biological properties which are considered pro-inflammatory, and they act on the same receptor, IL-1R1.

IL-1α mediates the early phase of inflammation, and it is present as a precursor in astrocytes, which is released when cell death via necrosis occurs [63]. It is then immediately active and behaves as an alarmin for the inflammatory cascade of cytokines and chemokines to begin. IL-1β is also a precursor but requires cleavage with caspase-1 to release the activated form into the extracellular space [63]. It is produced by haematopoietic cells such as blood monocytes, macrophages, dendritic cells and brain microglia. Both play a role in the adaptive immune response by enhancing the function of B and T cell subsets and activate neutrophils, monocytes and macrophages as part of the innate immune response [64]. The IL-1R1 receptor can be blocked by binding of IL-1ra which inhibits the actions on IL-1 and therefore potentially has an anti-inflammatory effect [65, 66]. IL-1 activity can also be reduced by binding it to an alternate receptor, IL-1RII, which is a decoy receptor with no onward signalling [67].

When assessing the cerebral response to TBI, IL-1α and IL1-β and their receptor activation have been considered important. Mouse studies have demonstrated that inhibition of IL-1β can result in reduced microglial activation and infiltration with neutrophils and T cells, which may relate to reduction in hemispheric tissue loss and cognitive decline [68]. This is supported by human TBI studies which have correlated high levels of IL-1ra with a more favourable neurological outcome than patients with low levels [66]. The IL-1ra/IL-1β ratio was also significantly higher in patients with a favourable outcome, and it is suggested that this ratio may relate to the balance between the patients’ pro- and anti-inflammatory state. Early clinical and pre-clinical studies have supported IL-1ra as a potentially protective treatment against brain injury from conditions such as stroke and subarachnoid haemorrhage, which drives continued interest in this molecule [69].

Despite the elements of traumatic head injury and inflammation in CSDH, there has been relatively little focus on IL-1. Only one study has measured IL-1β, and perhaps surprisingly, the levels were significantly lower in CSDH fluid when compared with serum [62]. This study also looked at IL-1ra and found no significant difference in levels when comparing serum and CSDH fluid. No studies have reviewed IL-1a. Given their role in TBI, the IL-1 family and its relative concentrations may be of interest in CSDH, and further work is needed to explore this.

### Interleukin-6 and -8

Interleukin-6 and -8 (IL-6 and IL-8) are being reviewed together because in many conditions their production is co-ordinated, possibly due to a common signalling pathway [70].

IL-6 is an important inflammatory cytokine secreted by a variety of cell types including fibroblasts, monocytes and endothelial cells [71]. It has a key role in the acute response to inflammation, promoting B and T cell differentiation, platelet production, acute phase protein induction and can enhance leucocyte recruitment by upregulating chemokines adhesion molecules [70, 71]. It is released in response to soft tissue trauma and haemorrhage, and dysregulated IL-6 has been linked to autoimmune diseases and cancer [70, 72]. It is also recognised to have some neurotropic and neuroprotective effects, particularly within the field of TBI [43, 71]. Many papers have highlighted significant increases in levels of IL-6 in CSDH fluid compared with peripheral blood [53, 62, 73, 74]. Activation of the Janus kinase-signal transducer and activator of transcription (JAK-STAT) signalling pathway has been identified in fibroblasts in the CSDH external membrane and is regulated by several cytokines, including the highly abundant IL-6 [75, 76]. Particularly dominant levels of activated STAT3 are seen within the fibroblasts and endothelial cells and are fundamental for cell growth, therefore implicating IL-6 as a potential driver/activator for ongoing CSDH membrane growth.

IL-8 is from a family of small, polypeptide cytokines that have pro-inflammatory and reparative properties [77]. It is classically recognised as a neutrophil chemoattractant, with IL-8 acting as a stimulus to draw neutrophils, and other inflammatory cells carrying the IL-8 receptor, to the site of IL-8 release [77]. Conversely, it has been shown to act in an anti-inflammatory manner, limiting the extent of leucocyte adhesion to vessel walls at sites of inflammation [78]. It is certainly known to be able to modulate its own receptor expression, and this may be important in controlling response to inflammation. More latterly, IL-8 has also been recognised to play a key role in angiogenesis, with production from endothelial cells, leucocytes and fibroblasts resulting in capillary tube formation, endothelial cell proliferation and MMP-2 release [79].

Both IL-6 and IL-8 have been identified as being significantly elevated in CSDH fluid [53, 61, 73, 74, 80]. More significantly, high levels of these cytokines have been correlated with increased risk of CSDH recurrence and the related imaging features of recurrence [53, 73]. As discussed, whilst these cytokines can be considered pro-inflammatory, they also have protective functions. Therefore, it is difficult to know whether high levels in patients with recurrence signifies that they are the driving force for CSDH expansion or whether they are indicative of the protective response occurring in relation to injury from another mechanism. A lower risk of recurrence has been correlated with increased levels of anti-inflammatory cytokines (e.g. IL-10) [61, 62]. Therefore, overall cytokine profiles certainly appear useful in being able to predict the risk of recurrence. Cytokine patterns have also been correlated with the time interval since injury, and it may be this, and hence the stage of the inflammatory cycle, that is actually important in predicting recurrence [61, 62].

### Interleukin-10

Considered an anti-inflammatory cytokine, interleukin-10 (IL-10) has a role in deactivating T cells, monocytes and macrophages and reducing subsequent pro-inflammatory cytokine production (e.g. IL-1) [81, 82]. In one study on CSDH patients, the majority were found to have low (<60 pg/mL) rather than high (>200 pg/mL) concentrations of IL-10 [83]. The lower concentrations were also associated with lower concentrations of IL-6 and -8, suggesting there is a reciprocal response of anti- to pro-inflammatory cytokines in the body’s attempt to control inflammation. Some reports have suggested consistently raised levels of IL-10, as well as the anti-inflammatory cytokine IL-13, when comparing CSDH fluid with serum [61, 74]. As cytokines are known to vary in their temporal profile, the likelihood is that levels will vary between patients depending on the stage of inflammation at the time of sampling. Different inclusion criteria for studies and time patterns from trauma to surgery might be important. Observing the relative levels of cytokines at more than one time point would be helpful in clarifying the patterns occurring.

### Other cytokines

A range of other pro-inflammatory cytokines have been shown to be significantly raised in CSDH compared with serum: IL-2R, IL-5, IL-6 and IL-17 [61], whilst others have been shown to have reduced levels: TNF-α, IL-1β, IL-2 and IL-4 [61]. Several chemokines are also found in high concentrations in CSDH fluid: chemokine ligand 2 (CCL2, also called MCP1), chemokine ligand 9 (CXCL9) and chemokine ligand 10 (CXCL10, also called interferon gamma-induced protein 10 or IP-10) [61]. These chemokines are similar to IL-8, as they act as potent chemoattractants and angiostatic factors. Eotaxin-3 is a chemokine with a specific role for attracting eosinophils and is important in contributing to the fibrosis contributing to CSDH membrane growth [18].

## Pharmacotherapy

For patients who become symptomatic from CSDH, which can range from headaches to neurological deficits and finally coma, surgery is often considered the mainstay of treatment. In cases where patients are critically unwell, they require emergency evacuation of the haematoma to resolve the acute decompensation due to raised intracranial pressure. However, there is a large cohort of patients who are clinically stable with appearances of CSDH found on imaging who would be appropriate for a more conservative treatment pathway, such as a drug therapy to help resolve the haematoma. Further to this, in those patients who are treated surgically, approximately 11% will have a recurrence of the collection necessitating further surgery [84]. Therefore, there is a call for a drug therapy which could help prevent this from happening, thus avoiding multiple surgeries for CSDH. Scientists have sought to discover drugs which influence the pathophysiological factors already discussed, in the hope they can provide a conservative and potentially preventative treatment option for CSDH. These therapies will be further discussed here.

### Dexamethasone

Steroids have long been used in the context of CSDH, and in 1974, it was summarised that steroids aid in the resolution of CSDH, supporting medical management rather than surgery in some cases [85]. Dexamethasone is a synthetic version of naturally occurring corticosteroid hormone and was first made in 1958 and heralded as a potent anti-inflammatory drug [86]. In 1976, Glover and Labadie reported that dexamethasone appeared to cause the formation of significantly smaller and lighter blood clots in CSDH, without the presence of a capsule [87]. They suggested that dexamethasone inhibited the inflammatory response and hence proper membrane development, the latter of which is essential as a source of continued haemorrhage for clot CSDH growth.

In 2005, a study on 112 CSDH patients assessed the role of dexamethasone, with a clear trend of reduced recurrence following surgery in those given dexamethasone versus surgery alone [88]. Further to this, 25 out of 26 patients had complete resolution of the CSDH following conservative management with dexamethasone only. Several other studies have supported these findings with reductions in recurrence in surgical patients or successful conservative management of CSDH with dexamethasone therapy [89–92]. By reducing recurrence, dexamethasone could significantly reduce mortality, particularly if given for longer time periods pre-operatively; however, further level 1 data is needed to support this therapy [93, 94].

It is well recognised that the anti-inflammatory effect of corticosteroids is mediated through gene expression, altering transcription of inflammatory proteins such as cytokines and chemokines [95–98]. Other signaling pathways involved in inflammation and membrane function are also effected, and there is a significant effect on differentiation and function of immune cells, such as B and T cells, dendritic cells and macrophages [95, 96, 99]. Some clinical studies have identified mediators that reflect anti-inflammatory mechanisms occurring in the brain, such as tuberculous meningitis patients showing decreased MMP-9 levels in CSF following administration of corticosteroids [100]. Cerebral extracellular fluid (brain microdialysate) in brain tumour patients receiving dexamethasone has also shown significantly increased concentrations of the anti-inflammatory IL-1ra and TIMP-1 [101].

Despite the role of dexamethasone as an anti-inflammatory, since the 1960s, its primary application in neurosurgery has been in the treatment of cerebral oedema [102]. This has particularly focused on cerebral oedema secondary to brain tumours but also includes oedema in relation to head injury and haemorrhage [102, 103]. Further to this, corticosteroids have also been used to reduce oedema in conditions such as spinal cord compression and high-altitude pulmonary and cerebral oedema [104, 105]. The suggested mechanism is a direct action on the vascular endothelium, leading to a reduction in vascular permeability and hence fluid accumulation around brain tumours [106–108]. For brain conditions in general, steroids can reduce the permeability of the BBB, by modifying capillary endothelial cells and regulation of tight junctions via expression of the occludin gene [109]. This all leads to “tightening” of the BBB and hence difficulty for fluid, but also other inflammatory cells such as leucocytes, to enter the brain.

It is plausible that dexamethasone has the same effect on the vascular endothelium of the “leaky” blood vessels seen in CSDH membranes, thereby reducing fluid exudation and bleeding and allowing resolution of the collection. None of these theories have been tested on a molecular level in CSDH patients, and therefore, the true mechanism of action of dexamethasone in this pathology remains unknown. One of the major drawbacks of steroid therapy is the significant side effect profile seen with systemic application, a challenge which may outweigh the benefits of its use [110, 111]. The CSDH population, who are largely elderly, may be particularly at risk of certain side effects of corticosteroids such as diabetic complications, ocular hypertension and open-angle glaucoma [112, 113]. Brain tumour studies have shown that the toxic effects of dexamethasone are dose-related, and lower daily doses (4 mg compared to 16 mg) and courses shorter than 28 days reduce this risk significantly [114]. Dosing must also take into consideration the pharmacokinetic and dynamic properties of dexamethasone. It has very good oral bioavailability (76–90%) and high potency but a relatively short plasma half-life of 4–4.7 h [95, 115]. It only takes 1–1.5 h to reach its peak plasma concentration, hence the rapid clinical effect, but despite a short plasma half-life is considered a long-acting steroid with a biological half-life of 36–54 h [95, 116]. Several trials to establish the evidence for dexamethasone efficacy, but also appropriate administration and dosing, in CSDH treatment are ongoing throughout the world [117–119].

### Atorvastatin

Although classically used as a cholesterol-lowering drug, laboratory experiments have shown that atorvastatin has a range of other properties which are relevant in CSDH. This includes anti-angiogenic effects (inhibiting VEGF and IL-8), anti-inflammatory effects (reducing TNF-α and MCP1) and even fibrogenic effects by reducing collagen deposition [120, 121]. These wide-ranging effects make it difficult to know the exact mechanism at work in CSDH treatment. Preliminary studies have suggested efficacy of atorvastatin as a conservative treatment option in CSDH with complete haematoma resolution within 6 months in two studies, although patient numbers only totaled 31 [122, 123]. Reduced need for surgical intervention with atorvastatin was also identified by Chan et al. in a study on 24 patients [124]. This was supported by a prospective placebo-controlled trial on 80 CSDH patients showing reduction in surgical intervention in patients on atorvastatin; however, the paper was subsequently retracted with a damning admittance that the results were misleading and exaggerated with serious errors in data analysis [125, 126]. Further to this, the same group published data suggesting atorvastatin could reduce CSDH recurrence after initial surgery but subsequently retracted this paper, leading to further uncertainty about the true efficacy of atorvastatin [127, 128]. Results from a large-scale, multi-centre, randomised study are currently awaited and will hopefully provide a more robust answer [129].

### Angiotensin-converting enzyme inhibitors

There is evidence that inflammation and angiogenesis play important roles in atherogenesis (the formation of atheromatous plaques) in coronary disease [130]. Treatment with angiotensin-converting enzyme (ACE) inhibitors has been found to reduce plaque vulnerability and rupture by regulating inflammatory cells and neovascularisation. It is therefore understandable that ACE inhibitors are postulated to play a role in reducing CSDH recurrence, through mediation of angiogenesis, possibly via VEGF [131]. However, a prospective randomised study on 47 CSDH patients comparing 5 mg perindopril daily with placebo for 90 days post-operatively was stopped early due to failure to show any difference [132]. It is important to highlight that there was a large list of exclusions in this study and that they did not see any recurrences in the 47 patients randomised, but a recurrence rate of 17.6% in those excluded. This suggests they may have been excluding those patients at highest risk of recurrence and hence not seeing any benefit with the ACE inhibitor treatment. Further research is needed to elucidate whether atorvastatin offers any benefit in CSDH, and this published study serves as a reminder that trials must be as pragmatic as possible to avoid selection bias and unreliable results.

### Tranexamic acid

Due to the hyperfibrinolytic state in CSDH, it follows that an anti-fibrinolytic drug such as tranexamic acid would help with resolution of the haematoma accumulation. A recent trial has already supported its clinical utility, showing reduction in death due to haemorrhage in acute trauma patients given tranexamic acid [133]. One small study reviewed 21 CSDH patients, 3 treated with a combination of surgery and tranexamic acid and 18 with tranexamic acid alone [134]. All patients had complete resolution of their CSDH with no recurrences, following varying lengths of drug treatment. There is now a prospective trial in progress assessing tranexamic acid in CSDH management, and the results of this are keenly awaited [135].

## Conclusions

Overall, it is clear that there are multiple drivers promoting expansion of a CSDH. Following trauma, which is often minor and not always evident, there appears to be a complex process of interrelated mechanisms including inflammation, membrane formation, angiogenesis and fibrinolysis that propagate an increase in CSDH volume. Many different molecular cascades occur throughout CSDH progression, and it is unlikely that there is a single prime pathway. However, certain molecules such as IL-6 and -8 do appear to be valuable in helping predict recurrence. The highly vascular and highly permeable outer membrane is evidently a source of inflammatory mediators as well as regular bleeding. The subsequent array of molecules found within the CSDH fluid provide evidence for a role of localised hyperfibrinolysis and continuous inflammation contributing to the haematoma expansion. This understanding has helped to guide the development of therapies that have the potential to modulate CSDH growth, such as anti-inflammatory drugs (dexamethasone) and anti-fibrinolytic drugs (tranexamic acid). Further to this, if we can understand the molecular patterns in the CSDH haematoma, then we may be able to tailor certain treatments to specific high-risk patients in the future. For example, those with very high levels of IL-6 and -8 may be more responsive to certain anti-inflammatory treatments and/or need a longer course of treatment than those with lower levels. The wide range of markers and their apparent difference between patients mean that there is unlikely to be a one-size-fits-all treatment for CSDH. The ultimate aim should be that through good understanding of the underlying molecular biology, focused and specific treatment algorithms for CSDH can be developed.

## Abbreviations

Ang: Angiopoietin
BBB: Blood-brain barrier
CCL/CXCL: Chemokine ligand
CSDH: Chronic subdural haematoma
FDP: Fibrin/fibrinogen degradation product
HIF: Hypoxia-inducible factor
IL: Interleukin
JAK-STAT: Janus kinase-signal transducer and activator of transcription
JNK: c-Jun N-terminal kinase
MAPK: Mitogen-activated protein kinase
MCP: Monocyte chemoattractant protein
MMP: Matrix metalloproteinase
NO: Nitric oxide
PGE: Prostaglandin E
PI3-Akt: Phosphatidylinositol 3-kinase-serine/threonine kinase
PICP: Procollagen type 1
PIIINP: Procollagen type 3
TBI: Traumatic brain injury
TNF: Tumour necrosis factor
tPA: Tissue plasminogen activator
VEGF: Vascular endothelial growth factor
